# Left ventricular structure and function following renal sympathetic denervation in patients with HFpEF: an echocardiographic 9-year long-term follow-up

**DOI:** 10.3389/fcvm.2024.1408547

**Published:** 2024-06-11

**Authors:** Alexander Vogt, Alexander Plehn, Carlo Atti, Michael Nussbaum, Jörn Tongers, Daniel Sedding, Jochen Dutzmann

**Affiliations:** ^1^ Department of Internal Medicine III, University Hospital Halle (Saale), Halle (Saale), Germany; ^2^ Praxisklinik Salzatal, Salzatal, Germany; ^3^ Department of Internal Medicine, Medical University Graz, Graz, Austria

**Keywords:** renal sympathetic denervation, heart failure with preserved ejection fraction, echocardiography, hypertension, long-term follow-up

## Abstract

**Background:**

High blood pressure is a major risk factor for cardiac remodeling and left ventricular hypertrophy, increasing cardiovascular risk and leading to heart failure with preserved ejection fraction (HFpEF). Since renal sympathetic denervation (RDN) reduces blood pressure in the long term, we aimed to investigate the long-term effect of RDN in patients with HFpEF in the present analysis.

**Methods:**

Patients previously enrolled in a local RDN registry who underwent high-frequency RDN with the use of the Symplicity Flex® renal denervation system between 2011 and 2014 were followed up. The patients were assessed by 24-h ambulatory blood pressure measurement, transthoracic echocardiography, and laboratory tests. We used the echocardiographic and biomarker criteria of the Heart Failure Association (HFA)-PEFF (Pre-test assessment, Echocardiography and Natriuretic Peptide Score, Funkctional testing, and Final aetiology) score to identify patients with HFpEF.

**Results:**

Echocardiographic assessment was available for 70 patients at a 9-year long-term follow-up. Of these patients, 21 had HFpEF according to the HFA-PEFF score. We found a significant reduction of the HFA-PEFF score from 5.48 ± 0.51 points at baseline to 4.33 ± 1.53 points at the 9-year follow-up (*P* < 0.01). This decrease was due to a greater reduction in morphological and biomarker subcategories [from 1.95 ± 0.22 to 1.43 ± 0.51 points (*P* < 0.01) and from 1.52 ± 0.52 to 0.90 ± 0.63 points (*P* < 0.01), respectively] than in the functional one. Morphologically, there was a reduction in left ventricular hypertrophy and left atrial dilation.

**Conclusions:**

The present analysis suggests that RDN may lead to a regression of the extent of HFpEF beyond a reduction in blood pressure and thus possibly contribute to an improvement in prognosis. More detailed information will be provided by ongoing randomized sham-controlled trials.

## Introduction

High blood pressure is a major risk factor for the development of cardiovascular diseases such as coronary artery disease, myocardial infarction, or stroke leading to premature death (1). Even before cardiovascular disease becomes clinically apparent, cardiac remodeling and left ventricular hypertrophy (LVH) may be detectable, increasing cardiovascular risk independently of blood pressure (2–4).

Hypertension, left ventricular remodeling, and hypertrophy can lead to ventricular dysfunction with elevated filling pressure (5). Elevated ventricular filling pressure, in turn, is associated with atrial remodeling and dilation, consequently leading to contractile dysfunction and arrhythmias (6). These structural and functional changes, among various others, contribute to heart failure with preserved ejection fraction (HFpEF) (7, 8). Treatment of arterial hypertension is thus the cornerstone of preventing ventricular hypertrophy and remodeling (9) and the development or progression of HFpEF (10, 11).

In addition to lifestyle changes and antihypertensive medication, renal sympathetic denervation (RDN) has emerged as a promising treatment modality, especially in patients with uncontrolled hypertension. RDN has been shown to effectively lower blood pressure, as demonstrated in several sham-controlled studies both with and without concomitant antihypertensive medication (12–16). Multiple registries have shown this effect to be sustained (17–19).

It can, therefore, be assumed that RDN also exercises an influence on cardiac remodeling and HFpEF. The aim of this study is therefore to investigate the long-term effect of RDN on cardiac morphology and function in patients with HFpEF.

## Methods

### Study population

**Figure 1:**
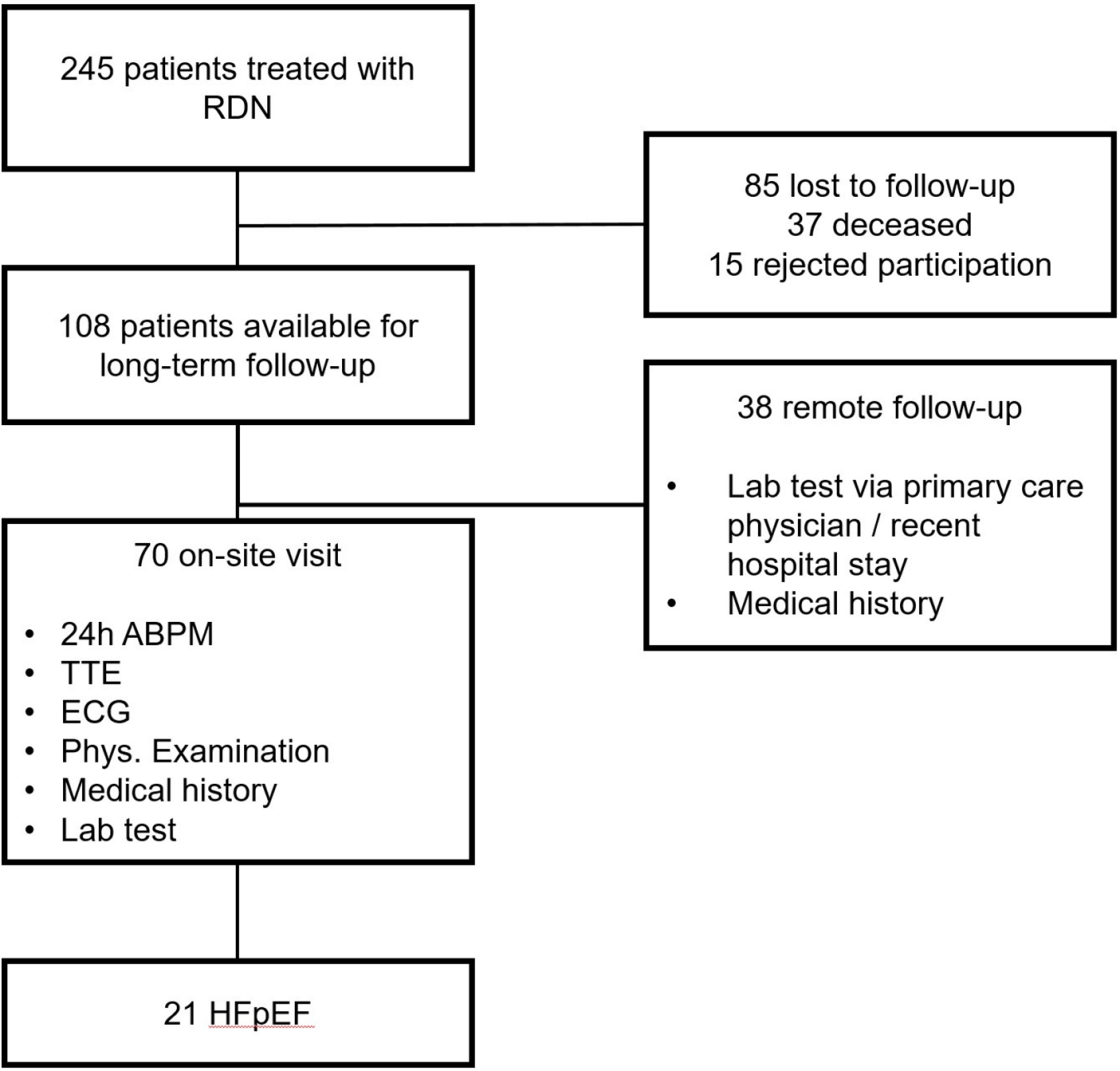
Study overview.

A total of 245 patients enrolled in the local RDN registry (www.drks.de; identifier: DRKS00004173) were contacted for a 9-year long-term follow-up (FU). All patients underwent radiofrequency RDN at the University Hospital Halle (Saale), Germany, between 2011 and 2014. They also underwent baseline evaluation by 24 h ambulatory blood pressure (ABP) measurement, laboratory tests, and transthoracic echocardiography (TTE) prior to renal denervation. A total of 108 patients were available for the 9-year FU. Full TTE data were accessible for 70 of these patients at the FU, and 21 patients were considered to have HFpEF according to the Heart Failure Association (HFA)-PEFF (Pre-test assessment, Echocardiography and Natriuretic Peptide Score, Funkctional testing, and Final aetiology) score (Figure 1) (7).

This study was approved by the local ethics committee. All patients provided informed consent.

### Renal denervation procedure

Technical and procedural details of the ablation systems have been described elsewhere (20). The procedure was performed by a single experienced operator (AP) using the Symplicity Flex® (Medtronic, Inc., Santa Rosa, CA, USA) renal denervation system following the instructions for use and recommendations of the device manufacturer. Accessory renal arteries were treated if the length and diameter were suitable.

### Transthoracic echocardiography

Transthoracic echocardiography was performed by experienced operators at baseline and during the 9-year FU using Vivid 7, Vivid 9 (GE Healthcare GmbH, Düsseldorf, Germany), and iE33 (Koninklijke Philips N.V., Amsterdam, Netherlands) ultrasound systems. Echocardiographic studies were done as recommended by the American Society of Echocardiography and the European Association of Cardiovascular Imaging (21) and digitally stored on a workstation. The studies were subsequently analyzed by two experienced echocardiography examiners (AV and CA) blinded to all other information.

Left ventricular (LV) dimensions were obtained by using the M-mode perpendicular to the LV long axis measured at the level of the tip of the mitral valve leaflets as well as using the biplane summation of disks method in apical two-and four-chamber views. LV ejection fraction (LVEF) was calculated by LVEF = (LVEDV − LVESV)/LVEDV based on the measurements obtained by the biplane summation of disks method. LV mass was calculated by using the formula LV mass = 0.8 × 1.04 × [(IVSd + LVIDd + LVPWd)^3^ − LVIDd^3^] + 0.6 g based on the measurements from the M-mode, as described above. The body surface area (BSA) was calculated as BSA = (body height × body weight/3,600)^1/2^. The left ventricular mass index (LVMI) was calculated as LV mass/BSA. The patients were divided into three groups based on left ventricular mass index (LVMI) (severe hypertrophy: LVMI ≥149 g/m^2^ for men and ≥122 g/m^2^ for women; moderate hypertrophy: LVMI >115 to <149 g/m^2^ for men and >95 to <122 g/m^2^ for women; no hypertrophy: ≤115 g/m^2^ for men and ≤95 g/m^2^ for women).

Relative wall thickness (RWT) was calculated as 2 × LVPWd/LVIDd. Left atrial (LA) volume was measured by the disk summation method at the end of LV systole and indexed to BSA the same way as with LVMI. LA volume was categorized into severe dilation (>34 ml/m^2^), moderate dilation (29–34 ml/m^2^), and no dilation (<29 ml/m^2^).

Diastolic function was evaluated according to established criteria (7). Pulsed-wave Doppler of the mitral inflow was used to assess E- and A-waves and the deceleration time of the E-wave (EDT). Tissue Doppler imaging (TDI) with pulsed-wave Doppler at the level of septal mitral annulus was used to measure E' velocities and the calculation of the E/E' ratio. Peak velocity derived from tricuspid regurgitation was obtained by using continuous wave Doppler.

### Assessment of HFpEF using the HFA-PEFF score

The HFA of the European Society of Cardiology (ESC) developed the HFA-PEFF score as a diagnostic tool to identify and classify patients with heart failure, especially those with HFpEF (7). The score uses a stepwise approach that integrates clinical and echocardiographic tests as well as the functional test. The central component is a comprehensive echocardiographic evaluation of morphological and functional parameters, which results in a score of up to 6 points.

In this analysis, patients who had a baseline HFA-PEFF score of greater than or equal to 5 were considered to have HFpEF (7).

### Ambulatory blood pressure measurements and clinical evaluation at 9-year FU

For on-site 9-year FU patients, 24 h ABP readings were done using standardized techniques and validated equipment (Mobil-o-Graph®, AMEDTECH GmbH, Aue, Germany) according to guideline recommendations (10, 22). The equipment was applied on site and the patients were instructed to leave the system in place to measure a full day–night cycle. ABP and heart rate were measured in intervals of 20 min from 6 a.m. to 10 p.m. and in intervals of 30 min from 10 p.m. to 6 a.m. Electrocardiogram was recorded prior to the application of the ABP equipment. Dipping was defined as a reduction of SBP of >10% during the night interval compared with daytime.

Blood samples were drawn to determine natriuretic peptides, creatinine, urea, and HbA1c. Due to a change in the brain natriuretic peptide (BNP) assay in the local laboratory, the baseline values were given as BNP (pg/ml) and values at the 9-year FU as N-terminal proBNP (NT-proBNP) (pg/ml). To make these comparable, cutoff values of the HFA-PEFF score were used (BNP >35 pg/ml and NT-proBNP >125 pg/ml for patients with sinus rhythm, and BNP >105 pg/ml and NT-proBNP >240 pg/ml for patients with atrial fibrillation).

Antihypertensive medication was recorded and divided into nine classes [renin–angiotensin–aldosterone system (RAAS) inhibitors (ACE inhibitors, angiotensin receptor antagonists, and renin inhibitors), calcium channel blockers (CCBs), beta blockers, diuretics, mineralocorticoid receptor antagonists (MRAs), alpha-adrenergic blockers, centrally acting sympatholytics, direct-acting vasodilators, and other medications].

### Statistical analysis

Continuous symmetrically distributed variables are presented as means ± standard deviation and confidence intervals. Between-group differences were compared using a *t*-test, and baseline and 9-year FU differences were compared using a paired samples *t*-test. Median and the 25% and 75% quartiles were calculated to describe skewed variables. Between-group differences of these variables were compared using a Mann–Whitney U test, and differences between baseline and 9-year FU were compared using a signed-rank test. Normal distribution was studied using a Kolmogorov‒Smirnov test. Between-group differences in categorical variables were compared using a χ^2^ test, and McNemar's test was used to compare baseline and 9-year FU of those variables. All endpoints were analyzed exploratively.

Statistical significance was accepted at *p* ≤ 0.05. Statistical analyses were performed with SPSS Version 28 (IBM, Armonk, USA) and GraphPad Prism version 9 (GraphPad Software, San Diego, CA, USA).

## Results

### Follow-up and patient characteristics

A total of 70 of 245 patients treated with RDN and included in the registry were available for this analysis. A total of 85 patients were lost to FU, 15 declined participation, 37 were deceased (4 died of myocardial infarction, 2 of stroke, 1 of subarachnoid hemorrhage, 5 from malignancies, 1 from upper gastrointestinal bleeding, 1 from coronary artery disease in combination with severe aortic stenosis, and 23 had unknown causes of death), and 38 had no on-site visit with echocardiography (Figure 1). The reasons for patients refusing to participate or visit the site were mainly related to long journeys or immobility. In addition, the 9-year FU took place during the COVID-19 pandemic, and therefore, contact restrictions or the risk of infection were cited as further reasons. A total of 21 of 70 patients had HFpEF according to echocardiographic and biomarker criteria of the HFA-PEFF score (7).

At the time of the procedure, the patients were 64.5 ± 8.6 years old, and 12 (57.1%) were male.

The patients were mildly obese with a body mass index (BMI) of 29.3 ± 4.5 kg/m^2^. Chronic kidney disease (estimated glomerular filtration rate, eGFR <60 ml/min/1.73 m^2^) was present in only two (9.5%) patients.

A total of four (19.0%) patients had known coronary artery disease, and diabetes mellitus was present in eight (38.1%) patients. The patients were treated with 12.4 ± 3.2 ablations. Compared with patients without HFpEF, those with HFpEF had significantly higher BNP levels (81.0 IQR 39.5–125.5 vs. 26.0 IQR 16.0–39.0 pg/ml, *P* < 0.01) at baseline. Systolic blood pressure was lower in patients without HFpEF, but this was not statistically significant. The number of antihypertensive medications did not show any differences (Supplementary Table S1).

The median time to follow-up was 9.4 (IQR 7.3–10.1) years.

### HFA-PEFF score

**Figure 2:**
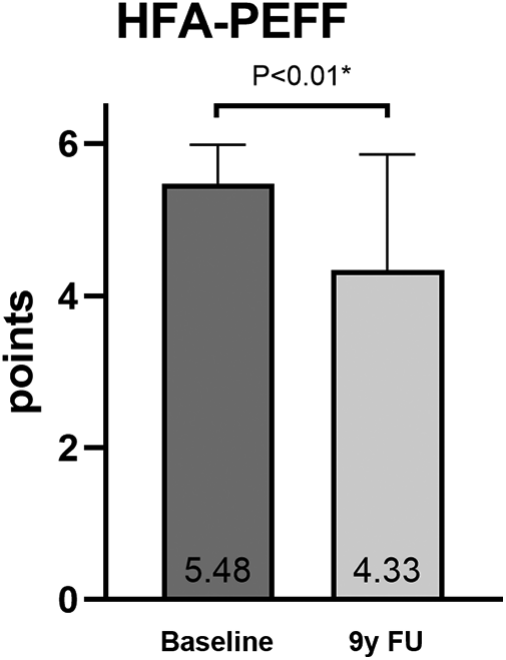
The HFA-PEFF score at baseline and at 9-year follow-up (comparison by using a paired *t*-test).

**Figure 3:**
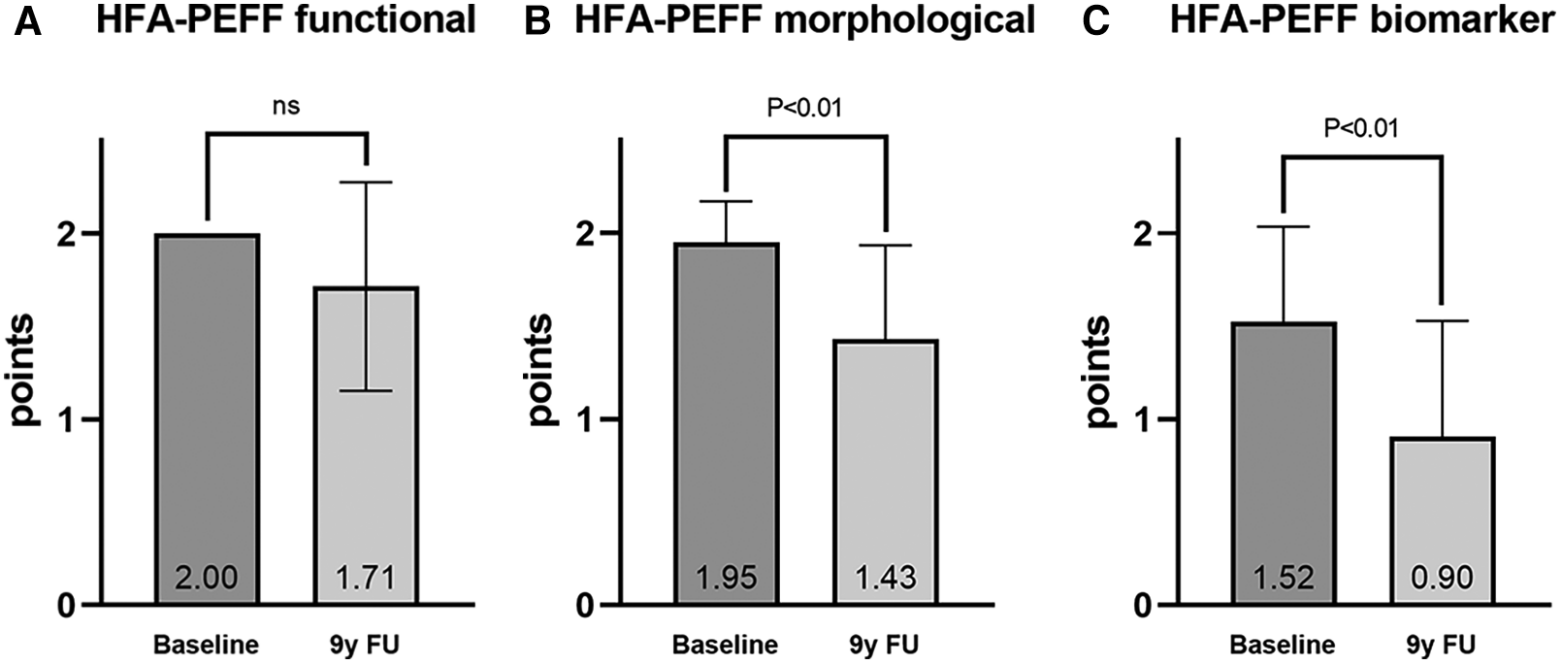
HFA-PEFF score subcategories: (**A**) functional, (**B**) morphological, and (**C**) biomarker (comparison by using a signed-rank test).

At baseline, the HFA-PEFF score was 5.48 ± 0.51 points and decreased to 4.33 ± 1.53 points at the 9-year FU (*P* < 0.01, Figure 2). This decrease was mainly driven by a reduction in the morphological and biomarker categories of the score. The points dropped from 1.95 ± 0.22 to 1.43 ± 0.51 (*P* < 0.01) in the morphological category and from 1.52 ± 0.52 to 0.90 ± 0.63 (*P* < 0.01) in the biomarker category. The points in the functional category showed a slight but non-significant decrease from 2.00 ± 0.00 to 1.71 ± 0.56 (Figures 3A–C).

Echocardiographic studies at baseline showed an LVMI ≥149/122 g/m^2^ in 17 (81%) patients. An LVMI of >115/95–<149/122 g/m^2^ was present in four (19%) patients.

**Figure 4:**
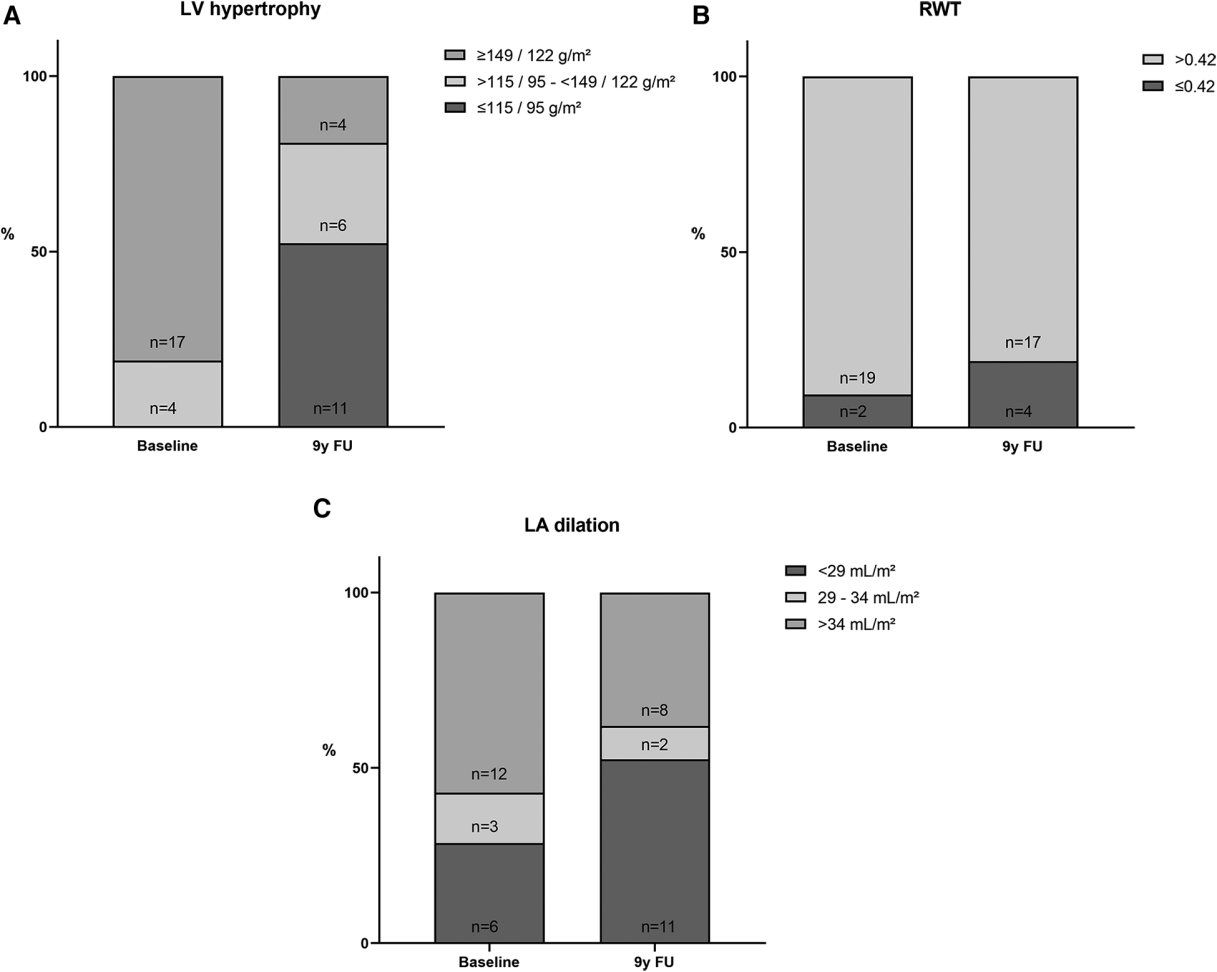
Distribution of LV hypertrophy (**A**), RWT (**B**), and LA volume index (**C**).

The number of patients with significant LV hypertrophy (≥149/122 g/m^2^) decreased considerably to 4 (19%) at the 9-year FU, whereas the number of patients without significant hypertrophy (≤115/95 g/m^2^) was 11 (52%, Figure 4A). A relative wall thickness >0.42 was present 19 (90%) at baseline and decreased slightly to 17 (81%) at the 9-year FU. The number of patients with significant LA dilatation (>34 ml/m^2^) decreased from 12 (57.1%) to 8 (38.1%), whereas the number of those without significant LA dilatation (<29 ml/m^2^) increased from 6 (28.6%) to 11 (52.4%, Figure 4C). Details on echocardiography can be found in Supplementary Table S2. Interestingly, the number of patients with a New York Heart Association (NYHA) functional class ≥2 increased numerically at the 9-year FU (2 vs. 6, *P* = NS).

### Blood pressure and antihypertensive medication

Mean systolic and mean diastolic blood pressure significantly decreased from baseline to the 9-year FU (systolic ABP from 152.2 ± 13.6 to 132.3 ± 13.7 mmHg, *P *= 0.013, and diastolic ABP from 86.3 ± 9.8 to 73.9 ± 10.0 mmHg, *P *< 0.01, Table 1). The calculated pulse pressure showed a clinically relevant decrease during the FU (65.9 ± 11.7–58.3 ± 11.4 mmHg, *P* = NS), although this was not statistically significant. The number of antihypertensive medications showed only a slight, non-significant decrease from 5.1 ± 1.3 to 4.9 ± 1.3. While the proportion of RAAS blockers, calcium antagonists, beta blockers, and diuretics remained constant, the proportion of centrally acting sympatholytics decreased (51.7% vs. 34.5%, *P *= NS). In contrast, the low proportion of MRA prescribed at baseline increased at the 9-year follow-up (9.5% vs. 33.3%, *P *= NS, Table 2).

**Table 1:** Patient characteristics.

Patient characteristics	Baseline	9-year FU	*P*
Age (years)	64.5 (±8.6)	73.1 (±8.0)	
Male, *n* (%)	12 (57.1)		
BMI (kg/m^2^)	29.3 (±4.5)	30.2 (±4.4)	NS
BSA (m^2^)	2.03 (±0.22)	2.04 (±0.21)	NS
Number of ablations	12.4 (±3.2)		
NYHA class ≥2, *n* (%)	2 (9.5)	6 (28.6)	NS
Atrial fibrillation, *n* (%)	0 (0.0)	5 (23.8)	NS
Blood pressure measurement			
24 h ABP sys (mmHg)	152.2 (±13.6)	132.3 (13.7)	0.013
Day	155.2 (±14.3)	135.1 (13.6)	<0.01
Night	145.7 (±16.6)	125.0 (±16.5)	NS
24 h ABP dia (mmHg)	86.3 (±9.8)	73.9 (±10.0)	<0.01
Day	89.3 (±10.6)	76.5 (±10.2)	<0.01
Night	80.6 (±9.3)	68.7 (±10.6)	NS
PP calc. (mmHg)	65.9 (±11.7)	58.3 (±11.4)	NS
Dipping, *n* (%)	8 (38.1)	6 (28.6)	NS
24 h heart rate (bpm)	59.0 (±6.7)	62.8 (±9.0)	NS
Laboratory			
Plasma creatinine (µmol/L)^+^	76.0 (62.5/87.0)	94.0 (75.5/114.5)	<0.01
Glomerular filtration rate (ml/min/1.73 m^2^)^+^	87.8 (73.0/125.5)	67.5 (52.5/81.0)	<0.01
BNP (pg/ml)^+^^a^	81.0 (39.5/125.5)	301.5 (204.0/536.5)	
BNP > cutoff, *n* (%)	21 (100)	19 (90.5)	NS
HbA1c (mmol/mol)	46.8 (±17.0)	44.0 (±11.0)	NS

bpm, beats per minute; PP, calculated pulse pressure.

BNP > cutoff, brain natriuretic peptide at baseline >35 pg/ml (sinus rhythm) and 105 pg/ml (atrial fibrillation); NT-proBNP at 9-year FU >125 pg/ml (sinus rhythm) and >365 pg/ml (atrial fibrillation).

^a^ Given as BNP (pg/ml) at baseline and NT-proBNP (pg/ml) at 9-year FU.

^+^indicates skewed variables.

**Table 2:** Antihypertensive medications.

	Baseline	9-year FU	*P*-value
ACEI/ARB/RI, *n* (%)	20 (95.2)	21 (100)	NS
CCB, *n* (%)	12 (57.1)	12 (57.1)	NS
Beta blockers, *n* (%)	20 (95.2)	18 (85.7)	NS
Diuretics, *n* (%)	18 (85.7)	16 (76.2)	NS
MRA, *n* (%)	2 (9.5)	7 (33.3)	NS
Alpha-adrenergic blockers, *n* (%)	8 (38.1)	10 (47.6)	NS
Centrally acting sympatholytics, *n* (%)	14 (66.7)	10 (47.6)	NS
Direct-acting vasodilators, *n* (%)	2 (9.5)	4 (19.0)	NS
Others, *n* (%)	14 (66.7)	12 (57.1)	NS
No. of antihypertensive medications	5.1 (±1.3)	4.9 (±1.3)	NS

ACEI, angiotensin-converting enzyme inhibitor; ARB, angiotensin receptor blocker; RI, renin inhibitors.

## Discussion

Arterial hypertension is one of the most common causes of HFpEF (5, 7, 23, 24). RDN is capable of lowering blood pressure both significantly and sustainably (12–16, 25, 26). It is therefore reasonable to investigate the possibility of a regression of HFpEF in patients after RDN. We chose the HFA-PEFF score (7) to categorize the patients in this study, as recommended by the 2021 ESC guidelines for the diagnosis and management of acute and chronic heart failure (27). It integrates functional, morphological, and biomarker criteria and thus allows to obtain a comprehensive view of HFpEF and its progression.

Our analysis of 21 patients with HFpEF out of 245 patients treated with RDN and included in our registry showed not only a significant reduction in blood pressure without an increase in the number of antihypertensive drugs but also a significant reduction of the HFA-PEFF score from 5.48 ± 0.51 to 4.33 ± 1.53 points. A reduction was also observed in all three sub-modalities of the score, which was significant in the categories of morphology and biomarkers. Notably, LA dilatation, but above all LV hypertrophy, was significantly reduced. This was previously shown by other research groups over a FU period of up to 12 months (28–31) as well as in a meta-analysis covering 17 prospective observational studies (32), which can possibly be explained by a reduction in the extent of cardiac fibrosis (33). Our analysis shows that this effect is sustained for a period of approximately 9 years.

In addition, there was a non-significant improvement in the functional parameters both in the individual values (see Supplementary Table S2) and in the corresponding score domain (Figure 3A). Conclusively, other authors were able to show a positive effect of RDN on individual echocardiographic functional parameters over FU periods of up to 12 months (28–31). Age as an independent risk factor for the development of HFpEF and age-related deterioration of functional echocardiographic parameters might be the reason why the HFA-PEFF score remained at 5 or 6 points in the 9-year FU in some patients in this study (Supplementary Figure S1) (34). Furthermore, there is evidence that early RDN is more likely to attenuate the progression of HFpEF than RDN performed later in the course of the disease, but we did not explicitly investigate this in our study (35). Similarly, the limitation of functional capacity in later life might have influenced the subjective wellbeing of some patients, and therefore, on average, there was an increase in NYHA class despite an improvement in the HFA-PEFF score. At the time of the 9-year FU, six of nine patients who had an HFA-PEFF score <5 points were treated with MRA, and therefore, this medication may have contributed to some of the observed echocardiographic benefits.

The amelioration of morphological parameters could provide a prognostic advantage, as a regression of LV hypertrophy *per se* is independently associated with an improvement in cardiovascular outcome (36–38). The same applies to LA dilatation, the regression of which also has a favorable effect on cardiovascular event rates (39, 40).

Since RDN also positively influences changes in sympathetic nervous activity (41), vascular stiffness (42, 43), and finally, ventriculo-arterial coupling (44), it appears to be a promising therapeutic approach in the treatment of HFpEF, the effectiveness of which is being investigated in the randomized, sham-controlled UNLOAD-HFpEF trial (NCT05030987), among others.

## Limitations

As is often the case in long-term registry studies, the absolute number of patients with HFpEF, as well as the proportion of all patients in the registry at the time of the 9-year FU, is relatively low. The willingness to participate may depend on the success of the treatment, and therefore, a selection bias may have arisen as a result. The small number of patients in this study also results in a reduction in statistical power, which might impact the findings. Furthermore, particularly in obese patients, it may be difficult to perform an accurate assessment of changes in cardiac systolic and diastolic function and volumes and, although in a given situation, the examiner concerned may be experienced, reliability with regard to both assessment and results may be questionable. In this study, the dosage of the medications was not recorded, and therefore, the effect of increases in dosage with a constant percentage of medication cannot be ruled out. As the RDN register was initially set up for the long-term study of the effects on blood pressure and the concept of HFpEF was not widespread at the time this study was designed, no stress tests were initially carried out. Patients with an HFA-PEFF score between 2 and 4 were, therefore, not differentiated more thoroughly and may not have been included in our analysis.

## Conclusion

The present analysis suggests that RDN may lead to a regression of the extent of HFpEF beyond the reduction in blood pressure and thus possibly contribute to an improvement in prognosis. More detailed information on this will be provided by ongoing randomized sham-controlled trials.

## Data Availability

The fully anonymized datasets analyzed in this study can be made available upon reasonable request after receiving approval from the ethics committee concerned. Requests to access the datasets should be directed to Alexander Vogt, alexander.vogt@uk-halle.de.
