# Supplementary material for: Left ventricular structure and function following renal sympathetic denervation in patients with HFpEF: an echocardiographic 9-year long-term follow-up
Source: Front Cardiovasc Med. 2024 Jun 11;11:1408547. doi: 10.3389/fcvm.2024.1408547 (PMC11196750; doi:10.3389/fcvm.2024.1408547)
Supplement: Supplementary file 1 [file Table1.docx]

**Supp. Table 1.** Baseline characteristics of patients with and without HFpEF

| **Baseline characteristics** | | | | | |
| --- | --- | --- | --- | --- | --- |
|  | | *All patients* | *HFA-PEFF ≥5*  *(n=21)* | *HFA-PEFF <5*  *(n=49)* | *P* |
| Age (years) | | 61.7 (±8.1) | 64.5 (±8.6) | 60.5 (±7.7) | NS |
| Male, n (%) | | 44 (62.9) | 12 (57.1) | 32 (65.3) | NS |
| BMI (kg/m²) | | 30.5 (±4.4) | 29.3 (±4.5) | 31.0 (±4.4) | NS |
| BSA (m²) | | 2.10 (±0.21) | 2.03 (±0.22) | 2.13 (±0.20) | NS |
| Number of ablations | | 12.2 (±2.6) | 12.4 (±3.2) | 12.1 (±2.3) | NS |
| *Blood pressure meassurement* | |  |  |  |  |
| 24h ABP sys (mmHg) | | 149.1 (±15.3) | 152.2 (±13.6) | 147.7 (±15.9) | NS |
|  | Day | 151.9 (±15.3) | 155.2 (±14.3) | 150.4 (±15.6) | NS |
|  | Night | 143.2 (±18.7) | 145.7 (±16.6) | 142.1 (±19.6) | NS |
| 24h ABP dia (mmHg) | | 86.5 (±11.5) | 86.3 (±9.8) | 86.5 (±12.2) | NS |
|  | Day | 89.3 (±11.7) | 89.3 (±10.6) | 89.3 (±12.3) | NS |
|  | Night | 80.3 (±12.6) | 80.6 (±9.3) | 80.1 (±13.9) | NS |
| Dipping, n (%) | | 20 (28.6) | 8 (38.1) | 12 (57.1) | NS |
| 24h heart rate (bpm) | | 64.2 (±10.0) | 59.0 (±6.7) | 66.6 (±10.4) | <0.01 |
| *Medical history* | |  |  |  |  |
| Coronary artery disease | | 8 (11.4) | 4 (19.0) | 4 (8.2) | NS |
| Atrial fibrillation | | 3 (4.3) | 0 (0.0) | 3 (6.1) | NS |
| Current Smoking | | 33 (47.1) | 9 (42.9) | 24 (49.0) | NS |
| Diabetes mellitus | | 26 (37.1) | 8 (38.1) | 18 (36.7) | NS |
| Chronic kidney disease (eGFR <60ml/min/1.73m²) | | 8 (11.4) | 2 (9.5) | 6 (12.2) | NS |
| # of antihypertensive medications | | 5.2 (±1.4) | 5.1 (±1.3) | 5.2 (±1.5) | NS |
| *Laboratory* | |  |  |  |  |
| Plasma Creatinin (µmol/l)^+^ | | 76.0 (65.8/89.3) | 76.0 (62.5/87.0) | 76.0 (66.5/90.0) | NS |
| Glomerular filtration rate (ml/min/1.73m²)^+^ | | 88.7 (77.2/100.7) | 87.8 (73.0/99.3) | 90.5 (78.5/100.7) | NS |
| BNP (pg/ml)^+^ | | 35.0 (21.5/70.3) | 81.0 (39.5/125.5) | 26.0 (16.0/39.0) | <0.01 |
| BNP > cut off^§^, n (%) | | 35 (50.0) | 21 (100) | 14 (28.6) | <0.01 |
| HbA1c (mmol/mol) | | 44.9 (±12.2) | 46.8 (±17.0) | 44.0 (±9.3) | NS |

ABP: ambulatory blood pressure, BMI: body mass index, bpm: beats per minute, BSA: body surface area, BNP > cut off: brain natriuretic peptide at baseline >35 pg/ml (sinus rhythm) and >105 pg/ml (atrial fibrillation), eGFR: estimated glomerular filtration rate. ^+^ Indicates skewed variables.

**Supp. Table 2.** Echocardiographic follow-up of patients with HFA-PEFF score ≥5

| **Echocardiographic follow-up, HFA-PEFF ≥5** | | | | | |
| --- | --- | --- | --- | --- | --- |
|  | *Baseline* | *12 Months* | *9y FU* | *P* | |
| *Dimensions* |  |  |  |  |  |
| LAVI (mL/m²) | 38.6 (±15.8) | 38.4 (±12.7) | 34.6 (±16.7) | NS | NS |
| IVSd (cm) | 1.32 (±0.28) | 1.33 (±0.21) | 1.26 (±0.27) | NS | NS |
| LVIDd (cm) | 4.83 (±0.73) | 4.60 (±0.66) | 4.58 (±0.97) | NS | NS |
| LVPWd (cm) | 1.42 (±0.25) | 1.46 (±0.33) | 1.25 (±0.38) | NS | NS |
| RWT | 0.61 (±0.20) | 0.65 (±0.17) | 0.59 (±0.30) | NS | NS |
| LVM (g) | 366.7 (±92.8) | 344.4(±109.1) | 223.3 (±65.5) | NS | <0.01 |
| LVMI (g/m²) | 170.8 (±41.0) | 166.2 (±43.7) | 108.0 (±27.6) | NS | <0.01 |
| LVEF (%) | 60.9 (±10.3) | 62.5 (±7.2) | 63.1 (±7.6) | NS | NS |
| GLS (%) | -15.7 (±3.1) | n/a | -13.9 (±3.5) | n/a | NS |
| FS (%) | 34.5 (±9.0) | 36.3 (±8.4) | 37.5 (±12.8) | NS | NS |
| TAPSE | 2.65 (±0.47) | 2.29 (±0.35) | 2.39 (±0.51) | NS | NS |
| *Doppler* |  |  |  |  |  |
| E (m/s) | 0.73 (±0.19) | 0.72 (±0.20) | 0.75 (±0.28) | NS | NS |
| E/A ratio | 1.01 (±0.31) | 0.92 (±0.21) | 1.47 (±1.07) | NS | NS |
| E‘ septal (cm/s) | 5.5 (±1.1) | 6.1 (±1.9) | 6.4 (±2.8) | NS | NS |
| E/E’ ratio | 13.9 (±3.7) | 14.0 (±8.8) | 12.6 (±4.2) | NS | NS |
| EDT (ms) | 235.8 (±74.6) | 223.1 (±63.5) | 218.2 (±59.5) | NS | NS |
| IVRT (ms) | 118.1 (±23.9) | 107.5 (±21.1) | 111.6 (±26.9) | NS | NS |
| TRV (m/s) | 2.27 (±0.71) | 2.35 (±0.80) | 2.23 (±0.50) | NS | NS |

**Supp. Table 3.** Echocardiographic follow-up of patients with HFA-PEFF score <5

| **Echocardiographic follow-up, HFA-PEFF <5** | | | | | |
| --- | --- | --- | --- | --- | --- |
|  | *Baseline* | *12 Months* | *9y FU* | *P* | |
| *Dimensions* |  |  |  |  |  |
| LAVI (mL/m²) | 37.8 (±11.7) | 37.4 (±9.7) | 30.1 (±12.6) | NS | <0.01 |
| IVSd (cm) | 1.33 (±0.30) | 1.37 (±0.24) | 1.34 (±0.30) | NS | NS |
| LVIDd (cm) | 5.02 (±0.86) | 4.77 (±0.77) | 4.76 (±0.84) | NS | NS |
| LVPWd (cm) | 1.31 (±0.28) | 1.43 (±0.35) | 1.25 (±0.45) | NS | NS |
| RWT | 0.54 (±0.17) | 0.63 (0.30) | 0.54 (±0.22) | NS | NS |
| LVM (g) | 386.0 (±139.5) | 368.9 (±89.8) | 251.2(±104.5) | NS | <0.01 |
| LVMI (g/m²) | 162.7 (±69.3) | 172.7 (±39.3) | 117.1 (±46.8) | NS | <0.01 |
| LVEF (%) | 61.7 (±8.4) | 64.4 (±8.0) | 59.7 (±11.5) | NS | NS |
| GLS (%) | -13.7 (±3.8) | n/a | -14.5 (±4.5) | n/a | NS |
| FS (%) | 34.7 (±9.0) | 37.6 (±10.0) | 34.2 (±12.1) | NS | NS |
| TAPSE | 2.63 (±0.47) | 2.56 (±0.49) | 2.47 (±0.52) | NS | NS |
| *Doppler* |  |  |  |  |  |
| E (m/s) | 0.75 (±0.18) | 0.77 (±0.19) | 0.77 (±0.22) | NS | NS |
| E/A ratio | 0.94 (±0.25) | 1.03 (±0.46) | 0.98 (±0.61) | NS | NS |
| E‘ septal (cm/s) | 6.9 (±2.0) | 7.4 (±2.4) | 6.9 (±2.0) | NS | NS |
| E/E’ ratio | 11.4 (±3.7) | 11.1 (±4.2) | 12.0 (±4.1) | NS | NS |
| EDT (ms) | 220.9 (±58.7) | 220.7 (±40.7) | 226.9 (±52.6) | NS | NS |
| IVRT (ms) | 108.7 (±26.8) | 106.1 (±26.4) | 100.5 (±22.0) | NS | NS |
| TRV (m/s) | 1.94 (±0.63) | 1.89 (±0.93) | 2.13 (±0.79) | NS | NS |
