# Supplementary figures and images for: Left ventricular structure and function following renal sympathetic denervation in patients with HFpEF: an echocardiographic 9-year long-term follow-up
Source: Front Cardiovasc Med. 2024 Jun 11;11:1408547. doi: 10.3389/fcvm.2024.1408547 (PMC11196750; doi:10.3389/fcvm.2024.1408547)

**Supp. Figure 1.** Distribution of individual scores at baseline and follow-up


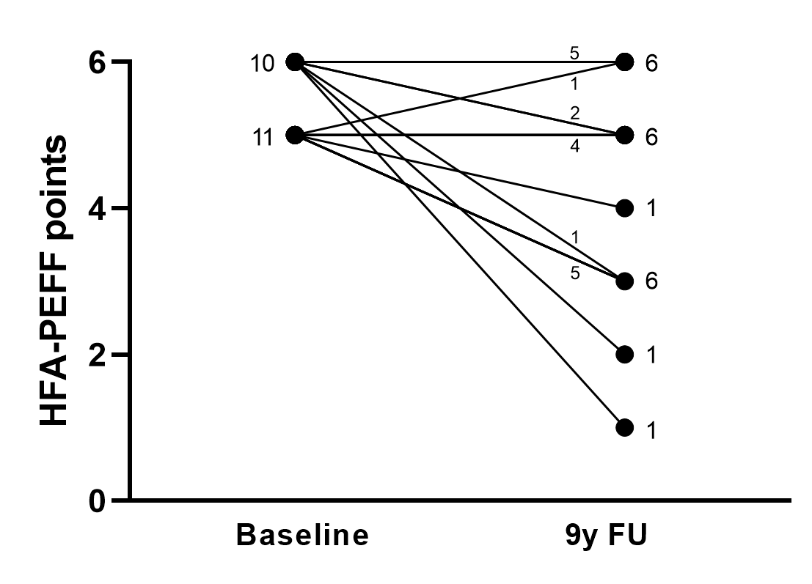

Supplement: Supplementary file 2 [file Datasheet1.docx]
